# Release of genetically engineered insects: a framework to identify potential ecological effects

**DOI:** 10.1002/ece3.737

**Published:** 2013-09-12

**Authors:** Aaron S David, Joe M Kaser, Amy C Morey, Alexander M Roth, David A Andow

**Affiliations:** 1 Department of Ecology, Evolution, and Behavior, University of MinnesotaSt. Paul, Minnesota, 55108, USA; 2 Department of Entomology, University of MinnesotaSt. Paul, Minnesota, 55108, USA; 3 Department of Forest Resources, University of MinnesotaSt. Paul, Minnesota, 55108, USA

**Keywords:** *Anopheles gambiae*, genetically modified organisms, homing endonuclease genes, malaria, population dynamics, problem formulation, risk assessment

## Abstract

Genetically engineered (GE) insects have the potential to radically change pest management worldwide. With recent approvals of GE insect releases, there is a need for a synthesized framework to evaluate their potential ecological and evolutionary effects. The effects may occur in two phases: a transitory phase when the focal population changes in density, and a steady state phase when it reaches a new, constant density. We review potential effects of a rapid change in insect density related to population outbreaks, biological control, invasive species, and other GE organisms to identify a comprehensive list of potential ecological and evolutionary effects of GE insect releases. We apply this framework to the *Anopheles gambiae* mosquito – a malaria vector being engineered to suppress the wild mosquito population – to identify effects that may occur during the transitory and steady state phases after release. Our methodology reveals many potential effects in each phase, perhaps most notably those dealing with immunity in the transitory phase, and with pathogen and vector evolution in the steady state phase. Importantly, this framework identifies knowledge gaps in mosquito ecology. Identifying effects in the transitory and steady state phases allows more rigorous identification of the potential ecological effects of GE insect release.

## Introduction

Genetically engineered (GE) insects have the potential to radically change pest and disease management worldwide. For example, there is great promise for GE technology to combat devastating insect-vectored human diseases, such as malaria, dengue fever, and chikungunya virus (Knols et al. 2007; Alphey et al. 2010; Lee et al. 2013). Release of GE organisms into the environment has generated considerable controversy regarding their potential ecological effects, and GE insects will not be an exception. Despite recent findings of low risk (USDA, United States Department of Agriculture 2008; see Murphy et al. 2010 for a risk assessment of *Wolbachia*-infected mosquitoes), approvals (USDA, United States Department of Agriculture 2008), and field trials (Harris et al. 2011; Lacroix et al. 2012) of GE insects, a general, synthetic, ecological framework is needed to guide the initiation of further ecological risk assessments (ERA). In general, GE organisms are not expected to cause any new kinds of ecological effects (e.g., Tiedje et al. 1989; Snow et al. 2005), but the potential ecological effects of these organisms must nevertheless be identified to determine possible adversity. In particular, new technological advances toward suppressing malaria-vectoring mosquitoes (e.g., Knols et al. 2007) provide a timely opportunity to revisit how such GE technology is evaluated.

Ecological risk assessment is a process used to evaluate undesired human-mediated environmental changes. ERA aims to combine estimates of the probability and magnitude of adverse ecological effects to predict risk, where adverse ecological effects are defined as undesirable changes that “alter important structural or functional characteristics or components of ecosystems” (EPA, US Environmental Protection Agency 1998). An ERA involves three main phases: problem formulation, analysis, and risk characterization. In the problem formulation phase, information is collected to determine the nature of the problem and identify the potential ecological entities at risk. Identifying the potential adverse ecological effects is a key step in this initial phase because it determines the scope of the ERA. Risk assessors can then conduct the analysis phase, where data are used to determine the likelihood and magnitude of potential effects, and also make comparisons among other management tactics and strategies (EPA, US Environmental Protection Agency 1998).

The first step toward identifying potential adverse ecological effects is to identify the potential effects associated with the implementation of a technology. Adversity can be determined later by stakeholders or through some other social process that takes into account the environmental priorities of people (e.g., Nelson and Banker 2007). Previous ERA approaches for GE insects have viewed the management goal (e.g., low pest density) as the state of the ecological system that should be assessed. In reality, transient states may arise during a GE release, and thus previous approaches may neglect evaluation of some ecological effects. Here, we provide a more comprehensive framework to identify the potential effects consistently and systematically for GE insects and help ensure future rigorous ERAs.

### Conceptual framework

A GE insect release may be thought of as an ecological perturbation whose ecological effects – adverse or not – occur in two phases: (1) a transitory phase during which the focal insect population (including the released GE insects) changes relatively rapidly in density, and (2) a steady state phase during which the population stabilizes at a constant density. The transitory phase begins when the dynamical behavior of the focal population changes (e.g., due to a deliberate or accidental release; an environmental change that allows an outbreak to occur; extinction of a consumer of the focal organism, etc.). The transitory phase will necessarily be associated with a temporary increase or decrease in insect density. The steady state phase begins when the focal population reaches a steady population density, which if the GE insect is effective, will be a new, suppressed population density. Perturbed populations could follow a number of pathways through these phases, some which represent effective implementation of GE insects and others which represent failed efforts or mismanaged populations using a variety of GE or non-GE tactics (Box 1).

**Box 1. Projected qualitative changes in relative population densities during the transitory and steady state phases of eight population scenarios**

Eight hypothetical population pathways depicting a transitory (unshaded region) and steady state phase (shaded region), and their potential evolutionary and ecological effects. Generally speaking, perturbed populations may increase and/or decrease during the transitory phase, resulting in a population density during the steady state phase that is higher, equivalent to, or lower than the initial density.

Below, we describe brief examples of each curve using an insect pest management tactic. Curves I–III represent three generalized pathways specific to current GE insect management techniques, and Curves IV–VIII represent additional pathways possible for a perturbed insect population. To simplify our presentation, we assume populations to have no age structure, although this should be considered in subsequent ERA steps.

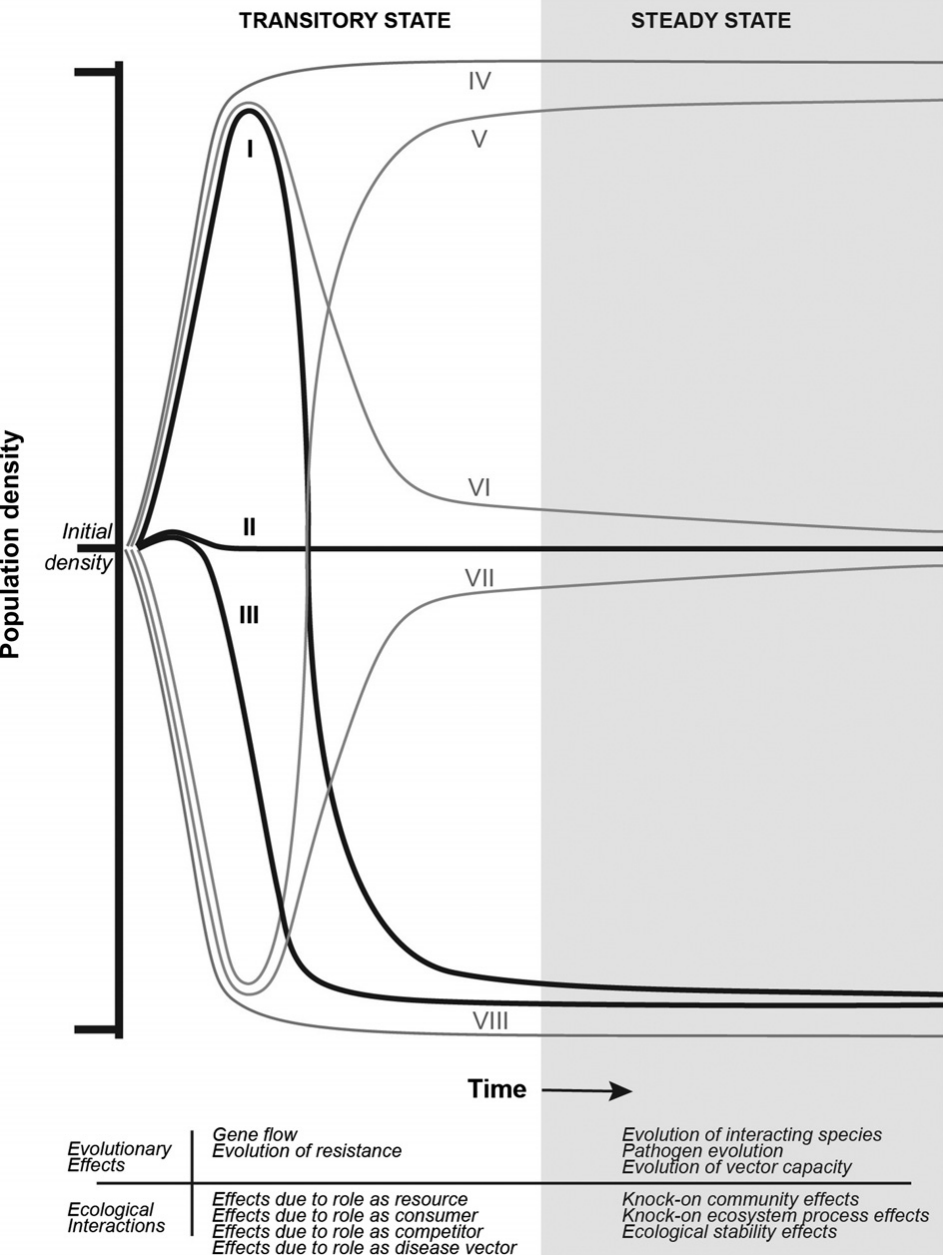

Curve I could describe the release of sterile insects, such as in the GE technique of Release of Insects with Dominant Lethal (RIDL; Ant et al. 2012), or non-GE sterile insect techniques (SIT). These strategies may cause a temporary increase in the focal population following mass release of sterile insects, but then result in a population crash. Similarly, successful classical biological control agent populations may follow this pathway by first increasing in density as individuals exploit the abundant food resource (target pest population) and reproduce, but then declining to steady low densities as they suppress the pest population.

Curve II could describe a GE insect technology that uses a strain-replacement mechanism (Gould et al. 2006); a released engineered strain eventually replaces a wild strain without altering the overall population density. Systems with strong gene drive mechanisms such as Wolbachia-infected mosquitoes (Dobson et al. 2002) or the proposed “killer-rescue system” (Marshall et al. 2011) would theoretically involve low release numbers and may result in little overall change in population density.

Curve III could describe the pathway of a GE insect containing a vector-suppressing homing endonuclease gene (HEG) (see Case Study and Box 2).

Curve IV could describe a released population, such as the biological control agent *Harmonia axyridis* (e.g., Koch et al. 2006), which has rapidly expanded beyond its intended purposes and remains at a high density.

Curve V could describe a case of pest resurgence following an insecticide application that, for example, also eliminates natural enemies (Hardin et al. 1995). Recent resurgence of the pestiferous brown planthopper (*Nilaparvata lugens*) is likely the result of resistance to insecticides that also negatively impact important natural enemies (Bottrell and Schoenly 2012).

Curve VI could describe a successful augmentative biological control agent in which a large population is released, but is then reduced back to its prerelease density following successful pest reduction (Hajek 2004).

Curve VII could describe a poorly managed pest population which was only temporarily suppressed and returns to the initial density. For example, the WHO malaria eradication campaign in Africa during the 1950s and 1960s saw initial success of vector suppression in some areas. However, myriad challenges led to the eventual failure of the program and the mosquito vector population returned (Webb 2011).

Curve VIII could describe successful traditional, non-GE pest suppression tactics, such as the use of chemical insecticides that immediately achieve and maintain suppression of the target population (Pedigo and Rice 2006).

We identify potential ecological and evolutionary effects of GE insects occurring during the transitory and steady state phases by finding effects stemming from any release or rapid change in density of an insect population. We draw on examples from a diversity of ecological circumstances, including other GE organisms, sterile insect technique, biological control, invasive species, and population irruptions. Casting a wide net over the literature allows us to overcome the limited data available for released GE insects by using lessons from other systems as a guide for identifying potential ecological effects of GE insect releases. Sterile insect technique provides examples although they are limited, because there have been few studies on ecological effects, except for efficacy of the control effort. Biological control provides many examples, because these are deliberate releases that are intended to have an ecological effect. Invasive species yield insights, because populations can change rapidly and both transitory and steadystate effects can be observed. Natural population irruptions also provide examples, because they are transient, many have been studied intensively, and ecological effects have been observed. We then apply this framework to a case study of the *Anopheles gambiae* wild mosquito – the predominant vector of malaria, currently being engineered to suppress the wild mosquito population. By considering the expected population pathway (Box 1), we are able to draw a subset of potential effects that we identified in the broader ecological literature.

Our framework is intended to help identify potential ecological effects associated with the release of a GE insect. The purpose of the framework is to identify all potential ecological effects for a specific GE insect release and not to compare management strategies. Importantly, our framework focuses on potential ecological and evolutionary effects without directly addressing adversity or any potential benefits of a release. Within the arena of risk assessment, this framework serves to support the Problem Formulation phase (EPA, US Environmental Protection Agency 1998); it provides the interested party with a novel, systematic approach to identify potential ecological effects. To complete an ERA, this must be followed by work to determine adversity and quantify the likelihood of exposure and/or magnitude of each potential effect.

## Transitory Phase

Two reasons to consider the transitory phase separately from the steady state phase are: (1) the transient effects may differ from steady state effects and (2) transient effects can in theory generate nontransient, persistent responses. Assumptions about the ecosystem prior to the release of the GE insect may not be valid during the transitory phase. After a release, densities of the focal insect may not immediately shift in the direction of the steady state density because the released GE insects cause the population to increase, and ecological and evolutionary effects propagating from these transient densities may differ from the steady state systems. For instance, Curve I in Box 1 shows a greater density in the transitory phase before ultimately achieving a steady state phase density that is lower than the initial density; Curve V shows a similar pattern in the opposite direction. Second, the ephemeral changes in density could generate ephemeral or long-lasting ecological or evolutionary changes in the ecosystem. The ephemeral changes may be distinct from those stemming from the steady state, and even though they may eventually disappear, their effects should not be assumed negligible.

### Evolutionary effects

#### Gene flow

Evolutionary effects may stem from transitory changes in gene flow. Gene flow, or the movement of genes from one population to another, can result from movement of individuals, gametes, or extracellular DNA segments (Slatkin 1985) and may occur within or between species (Whittemore and Schaal 1991). In cases where the population density significantly increases during the transitory phase (Box 1), an advantageous gene may more easily spread throughout a growing population because of the reduced influence of genetic drift and the higher number of potential migrants (Wright 1932). Conversely, decreases in population density (Box 1) could result in decreased gene flow due to a population bottleneck (Futuyma 1998; but see Sax et al. 2007). Some of these transitory changes in gene flow may carry over to the steady state phase. While gene flow among populations and subspecies may be desired in some cases of GE insect release (e.g., strain replacement technologies that seek to establish less pestiferous populations (Gould and Schliekelman 2004)), this is not always the case (e.g., Release of Insects Carrying a Dominant Lethal, RIDL® [Oxitec Ltd, Abingdon, UK] [Ant et al. 2012]) and does not negate the importance of considering effects of gene flow.

The impacts of intraspecific hybridization (i.e., admixture) are increasingly being discussed in the context of invasive species (e.g., Culley and Hardiman 2009). Admixture may have multiple effects, ranging from decreased fitness to heterosis (hybrid vigor) (Facon et al. 2011). Admixture has been observed in several insect species such as in the introductions of *Apis mellifera carnica* and *A.m. linguista* honeybees hybridizing with native *A.m. mellifera* populations in northwestern Europe (Jensen et al. 2005) and the introduction of African *Drosophila melanogaster* in American populations (Caracristi and Schlotterer 2003). In some cases admixture has had implications for management. For instance, novel genetic advantages to admixed individuals of the invasive ladybird beetle, *Harmonia axyridis,* have been implicated in the invasion success of the repeatedly introduced biological control agent *H. axyridis* in Europe (Facon et al. 2011). Examples of intraspecific gene flow also exist in the GE organism literature, primarily with respect to GE crops. Though key differences exist between GE crops and insect pests in their physiology (e.g., reproductive mechanisms) and purpose (e.g., cultivar improvements vs. pest control) (NRC, United States National Research Council 2002), GE crops still offer valuable insight into the types of effects that are possible for other GE organisms. Numerous studies have shown that intraspecific flow of the engineered gene has occurred between GE and non-GE varieties of several crops. For instance, transgenes from *Bt* corn cultivars were found in corn landraces in Mexico (Mercer and Wainwright 2008). Potential herbicide-tolerant transgenes in GE crops, such as rice, may spread to other varieties and enhance the fitness of a weedy conspecific by increasing resistance to herbivores or herbicides (Lu and Snow 2005). Conversely, if the crop transgene confers a fitness cost, transmission to wild populations could contribute to declines of small, isolated populations of wild plants (Haygood et al. 2003).

Transitory *inter*specific gene flow may occur through mating, hybridization, and introgression between GE and non-GE organisms. In particular, the presence of an engineered driver gene could make interspecific gene flow a concern, even in instances where the GE insect is endemic. Interspecific gene flow has been found between GE agricultural plants and wild plants (e.g., Zapiola and Mallory-Smith 2012). In natural systems, hybridization can produce many ecological consequences often resulting in strong negative impacts to native species (Kenis et al. 2009 and references therein). Hybridization between native gray ducks (*Anas superciliosa superciliosa*) and introduced North American mallards (*A. platyrhynchos*) has been implicated in population decline of gray ducks in New Zealand (Rhymer et al. 1994). Introgression of mtDNA between populations has been documented, and gene flow appears to have reduced phenotypic diversity and may ultimately lead to loss of the gray duck as a distinct species in New Zealand (Rhymer et al. 1994). Among insects, interspecific mating was a primary mechanism for the displacement of oriental green stink bug, *Nezara antennata,* by *N. viridula* in parts of Japan (Kiritani et al. 1963).

#### Evolution of resistance and loss of control efficacy

Perhaps the most commonly considered evolutionary effect is the evolution of resistance to a control tactic (e.g., insecticides). Insects continually acquire resistance to chemical insecticides (e.g., Hare 1990) and transgenic plants expressing insecticidal properties (Tabashnik et al. 2008). For GE insects, reduced control efficacy may also evolve through loss of the engineered gene in the population by such mechanisms as reduced genetic drive or selection against the GE individual owing to a fitness cost (Knols et al. 2007). The mechanism by which resistance or loss of control efficacy may occur depends on the particular tactic. In an SIT program directed against the melon fly in Japan, wild females evolved to reject mating attempts by the released sterile males (Koyama et al. 2004). In this case, the SIT program achieved eradication success by altering release numbers to counter evolved behavioral resistance. Although infrequently observed (Dyck 2005), other forms of behavioral variation could result in prereproductive isolation and thus could be selected for (e.g., melon fly exhibits allochronic variation in mating during the day) (Koyama et al. 2004). For RIDL, Alphey et al. (2011) showed that resistance to the dominant lethal gene is possible in some situations.

### Ecological interactions

In its interactions with other species, a GE organism may take on one of many ecological roles, such as resource, consumer, competitor, or disease vector. Changes in these roles during the transitory phase may lead to several potential ecological effects. For instance, during transitory increases in density, competition between the focal population and another species may intensify, while during transitory decreases in density competitors may be released from competition (Box 1). We draw on examples from natural population outbreaks and augmentation biological control to describe the scope of these potential effects.

#### Effects due to role as a resource

Changes in an insect population density due to GE release may affect those species that prey upon it. Increases in predator populations due to increased availability in prey have been documented in several natural systems, such as the spruce budworm (*Choristoneura fumiferana*). Spruce budworm populations periodically outbreak (Royama 1984), and these transitory increases in density have affected the bird populations that prey upon them (e.g., Venier et al. 2009). Passerine bird populations (Crawford and Jennings 1989) often increase, and several warbler species increased density more than 10-fold (Venier et al. 2009). In parallel, forest food webs responded to an increase in budworms with a greater diversity of higher trophic predators and parasitoids (Eveleigh et al. 2007).

While GE insect releases may not necessarily cause changes in biomass equivalent to spruce budworm outbreaks, augmentation biological control provides similar evidence that, even at levels achieved within the practical limits of insect rearing and release capabilities, increases in a resource population can have unintended consequences. In augmentation biological control, large numbers of an agent are released to supplement a naturally occurring population, which may increase populations of its predators. For example, several predator species such as carabid beetles (Snyder and Ives 2000) and convergent ladybird beetles (Colfer and Rosenheim 2001) preferentially feed on mummified aphids which contain developing parasitoid biological control agents (Brodeur and Rosenheim 2000). Much of the effect of the increased predator populations is to suppress the population of the augmented agent back to its initial density, and effects are likely to be transitory, ending once the agent has declined back to its original density. This may be caused by predators shifting their feeding preferences toward the superabundant agent population (Eveleigh et al. 2007). Attention should be given to whether a similar switching could mitigate the potential effects of an increased focal population after GE release, or if a dearth of predators may allow for continued increase of the focal population.

#### Effects due to role as a consumer

Release of a GE insect that persists during the feeding stages of its life cycle results in a transitory increase in a consumer population. Ecological theory suggests that an increase in a consumer population can lead directly to a decrease in a resource species, such as occurs during natural outbreak years of tree pests (e.g., Man and Rice 2010). For example, forest tent caterpillar (*Malacosoma disstria*) population outbreaks last 3–6 years and recur every 5–10 years, causing widespread defoliation of trees (Hildahl and Campbell 1975). The negative effects on tree populations are compounded during the later years of the outbreak, and may change forest compositions over time (Man and Rice 2010). Tree defoliation by *M. disstria* was associated with higher nest abandonment and partial loss of broods in black capped chickadees (Pelech and Hannon 1995). The potential for the focal population to switch resource/prey species during the transitory phase should also be considered as a possible ecological effect.

#### Effects due to role as a competitor

An initial increase (or decrease) in population size during the transitory state may suppress or displace (or release) a competitor species. Such effects are common among insect predators and parasitoids. Predators may compete via intraguild predation; superparasitism among parasitoids may result in interference competition, where the larvae of one species may suppress or kill the other (Brodeur and Rosenheim 2000). Competition among insect herbivores tends to be asymmetrical (Denno et al. 1995), and will be a more difficult problem for risk assessment.

#### Effects due to role as a disease vector

GE technology is in various stages of development to combat insect-vectored diseases, such as malaria and dengue fever, by engineering the vector to reduce the disease prevalence in humans. Changes in disease are dynamical in nature and may depend on the transient vector population. GE technologies are designed to reduce the disease transmission rate and the basic reproductive rate of the pathogen, *R*_0_, either by suppression of the vector population, or by replacement of the population with insects engineered to inhibit pathogen transmission.

In cases where the human population has built up acquired immunity from regular exposure to the vectored pathogen, reduction in transmission could lead to a reduction in prevalence of acquired immunity and herd immunity, which is the proportion of immune, noninfective individuals in a population (John and Samuel 2000). Loss of acquired immunity increases the susceptible population, which can lead to higher disease prevalence. High herd immunity may ‘interrupt’ the transmission of the pathogen by reducing the pathogen reservoir in the host population and the proportion of susceptible individuals. Acquired immunity reduces R_0_ by the proportion of noninfective immune individuals, *p* (i.e., herd immunity), so that the adjusted rate equals (1 − *p*)×*R*_0_ (Kyle and Harris 2008) and herd immunity reduces R_0_ directly. If immunity is maintained by repeated exposure to the pathogen, both types of immunity may be lost when vectors decline. As the vector population declines, *R*_0_ should decline. However immunity also declines, reducing *p*, and the adjusted pathogen reproductive rate may increase, potentially resulting in transient higher disease prevalence than if immunity were maintained at prerelease levels. The actual outcome of these dynamics, though, may be affected by the rates of change to populations of a given vector, pathogen, and associated immune populations.

The loss of herd immunity could be problematic if reduction of insect vectors is only temporary (e.g., Box 1 Curve VII). If the vector population rebounds and *R*_0_ increases faster than *p*, a temporary spike in disease incidence could occur. Reduced herd immunity has impeded efforts to eradicate or suppress other human diseases such as measles (Moghadas et al. 2008) and meningococcal diseases (Trotter et al. 2005). There is also evidence that reduced herd immunity may also impede efforts to suppress insect-vectored diseases through vector control. For example, dengue fever has seen a recent resurgence in Singapore where the disease had been suppressed for roughly 15 years. The upswing in disease is partially attributed to loss of herd immunity (Kyle and Harris 2008). During the reemergence of dengue in Singapore, there was an increased proportion of severe infections in adults as compared with children (Ooi et al. 2006).

## Steady State Phase

Assuming that the GE insect release was successful, the focal insect population should stabilize at a new steady state density after the transient changes in density. In this section, we identify potential ecological and evolutionary effects associated with a change in steady state population density. This phase is expected to last a long time relative to the transitory phase. In addition, although the focal population reaches a steady state, the ecosystem as a whole may lag behind the focal population. We identify evolutionary and ecological effects that may arise during the steady state phase by reviewing the literature on invasive species, sterile insect technique, biological control, and GE crops.

### Evolutionary effects

Evolutionary effects observed after some perturbation (e.g., extinction, invasion) reinforce the idea that changes in selection pressures can have broad effects for community genetics (Myers and Knoll 2001). Competitive release or introduction of novel mortality factors resulting from a GE insect release could have evolutionary consequences that would likely affect species differentially.

#### Evolution of interacting species

As a community adjusts to the changed focal population density, changes in the frequency of certain species interactions could result in novel selection pressures over short timescales. For instance, rapid evolution has been documented multiple times after species invasions (Sax et al. 2007). The Australian soapberry bug, *Leptocoris tagalicus*, has evolved mouthparts 5–10% longer over a 30–40 year period allowing it to feed on the seeds of the invasive balloon vine, *Cardiospermum grandiflorum*, (Carroll et al. 2005). Such rapid evolution might happen to an insect species released by the suppression of the focal species. Conversely, a GE insect release may extirpate the focal species from the region. Such local or global extinctions can have major evolutionary consequences. For example, the removal of certain avian pollinators has led to strong pollen limitation and consequential decreased reproduction in New Zealand forests (Anderson et al. 2011). Moreover, such pollinator suppression may have led to the evolution of traits favoring self-fertilization (Bodbyl Roels and Kelly 2011).

#### Pathogen evolution – virulence and transmission

GE insects that successfully suppress vector populations may alter the evolution of virulence and transmission of the vectored pathogen. Natural selection acts on two important levels for pathogens – (1) strains competing within a host affecting virulence, and (2) individual strains transmitted between hosts (May and Nowak 1995) – leading to a potential trade-off between these two levels. Virulence may be altered by a decrease in parasite load or within-host strain diversity, while transmission efficiency may be altered by a change in vector population density or parasite load (May and Nowak 1995; Bousema and Drakeley 2011). Because transmission of insect-vectored pathogens relies on infection of the vector, greater (or reduced) virulence in the noninsect host can be attained without affecting transmission efficiency (Ewald 2004). For instance, in a model of dengue virulence using different GE mosquito strategies, Medlock et al. (2009) found that virulence could increase or decrease with reduced vector density and transmission. Virulence and/or transmission rates of pathogens may evolve during the steady state phase, and these potential changes should be evaluated in context of public health goals.

#### Evolution of increased vector capacity

Vector capacity, the suitability of a vector species for transmission of the parasite, can constrain the evolution of virulence. In a study examining the bubonic plague vector, the rat flea (*Xenopsylla cheopis*), Lorange et al. (2005) suggested that poor vector capacity of the flea was a selective force resulting in higher pathogen virulence. The authors suggest that the combined effect of a low rate of infection (i.e., a low proportion of infected fleas are able to effectively transmit the pathogen) and high infection threshold (i.e., the pathogen must be present in a high enough dose for the flea to take it up when feeding) has resulted in a highly virulent pathogen that causes severe disease in humans. This result suggests that an inferior GE vector could select for a pathogen strain that confers higher or lower disease severity (Ewald 2011).

### Ecological interactions

A long-term reduction in insect abundance may have important consequences for other interacting species. These consequences may be readily observed when a species is removed or extirpated (Millennium Ecosystem Assessment 2005). Here, we consider potential knock-on effects, which are indirect or cascading effects resulting from a given perturbation of a new steady state on communities, ecosystems, and general ecological stability. As indicated below, knock-on effects commonly occur in ecosystems, and identifying them for any particular GE insect release requires sound ecological information about the community interactions and ecosystem functions of the focal species. This information rarely exists, but this should not be interpreted as the absence of potential knock-on effects.

#### Knock-on community effects

A long-term reduction in a focal species' density could release competitors, prey, or resources and allow them to increase and/or expand their range leading to such phenomena as trophic cascades or apparent competition (Snyder and Evans 2006). These effects themselves may affect additional species. For example, two species of gall flies (*Urophora affinis* and *U. quadrifasciata*) were introduced to control spotted knapweed (*Centaurea maculosa*). The gall fly populations have plateaued since introduction (Harris 1980) though the knock-on effects via their predators continue. Ortega et al. (2004) found that the increased fly population resulted in higher deer mouse densities because the additional nutrition provided by the fly larvae on knapweed seed heads increased mouse overwintering success. Furthermore, Pearson and Callaway (2006) showed that the elevated mouse population developed higher levels of hantavirus, a potentially fatal human pathogen. Knock-on effects can also occur when the focal population acts as a consumer. Invasive predators and detritivores commonly displace native species by diverse mechanisms, including exploitative competition, intraguild predation, shared natural enemies, or mating disruption (Snyder and Evans 2006).

The idea that community interactions change when population density differs is clearly illustrated by populations with multiple stable states. Gypsy moth (*Lymantria dispar*) population densities rapidly change between low and high stable states (Elkinton and Liebhold 1990). At low densities, populations in North America are regulated in part by small mammals that consume the immature stages. At high densities, however, mammals are unable to regulate these populations; instead gypsy moth populations are regulated by nuclear polyhedrosis virus (NPV), which kills gypsy moth caterpillars. Furthermore, change in NPV prevalence may have knock-on effects on other species. For example, parasitism rates of gypsy moth by the fly parasitoid *Compsilura concinnata* decreased when percent infection of NPV was higher (Hajek and Tobin 2011).

#### Knock-on ecosystem process effects

A change to a new steady state may also affect ecosystem processes. Many species typically contribute to multiple ecosystem processes, and a change in their density will alter these contributions. When a single species generates most of the ecosystem process (e.g., an effective classical biological control agent), such effects may be likely. However, most ecosystem processes involve the action of several to many species (e.g., pollination and decomposition), so a change in density does not necessarily result in a concomitant change in the associated ecosystem process.

Recent amphibian decline presents an example of how species population reduction may affect ecosystem processes. Due in part to the fungal disease chytridiomycosis, amphibian decline and extirpation has been rapid over the last two decades (e.g., Crawford et al. 2010). Tadpoles are important for maintaining the bioturbation of sediment, and their removal from aquatic systems decreases the resuspension of sediments and can lead to increased diatom biomass and decreased availability of algal foods used by other species, such as mayflies (Ranvestel et al. 2004).

The addition of a novel organism to a system can also affect ecosystem processes. The facilitation of the European earthworm invasion into North American forests by common buckthorn (*Rhamnus cathartica*) illustrates potential knock-on effects of the introduction of one species on whole ecosystem processes. Buckthorn is an exotic invasive shrub in the US which has established a high population density in many areas, providing nutrient-rich leaf litter and creating high shade conditions that cool soils (Heimpel et al. 2010). Importantly, these changes have facilitated a secondary invasion by European earthworms, which in turn has myriad effects on soil properties and nutrient cycling. Earthworms disrupt beneficial mycorrhizal interactions with native plants, increase the bulk density of soils, reduce the overall availability of nutrients like nitrogen and phosphorus, and increase the leaching of these nutrients from forest systems (Frelich et al. 2006). After adapting to the presence of earthworms, forest ecosystems often reach a new steady state where soil biotic communities and functions are significantly changed (Eisenhauer et al. 2011).

#### Ecological stability

A reduced density of the focal species may result in community destabilization. For example, plant–pollinator networks are generally thought to be stable due to the asymmetry and redundancy built into the networks (e.g., Bascompte et al. 2006). However, recent theory suggests that asymmetric networks may not be as stable as believed (Saavedra et al. 2011). Species that interact with the highest number of other species contribute the greatest to stability of plant–pollinator networks. These same species, however, are the most likely to go extinct. Although such networks may be fairly stable, they are precariously so, and if a highly interacting species is suppressed, this could lead to greater network instability (Saavedra et al. 2011).

Ecological systems may be influenced by hysteresis – the inability of a system to return back to its original state from an alternative state (Beisner et al. 2003). Hysteresis may hinder efforts to restore native vegetation in invaded habitats. For example, removal of invasive plants such as *Lonicera* spp. may actually increase exotic rather than native plant diversity (Love and Anderson 2009). A GE insect release could result in an undesirable steady state phase that cannot be easily reversed.

## Case Study: GE Mosquitoes for Malaria Control

Malaria is the fifth leading cause of death from infectious diseases worldwide and is particularly devastating in Africa, where the vast majority of deaths occur in children under the age of five (WHO, World Health Organization 2012). Malaria is a vector-borne blood infection caused by protozoan parasites in the *Plasmodium* genus (Bousema and Drakeley 2011) and is vectored to humans by *Anopheles* mosquitoes, most commonly by *An*. *gambiae* in Africa (WHO, World Health Organization 2012). Tactics such as DDT application and insecticide-treated bednets have been used to suppress malaria by reducing the mosquito vector population, but new GE technologies offer promising opportunities for greater efficiency and effectiveness of control.

One promising GE technology involves the use of homing endonuclease genes (HEGs) to drive a linked deleterious gene into the *An. gambiae* population and suppress the population (Box 2). A successful release of HEG mosquitoes may be expected to follow Curve III (Box 1) because the population increases only slightly during the transitory phase following release, then decreases until ultimately stabilizing at a low density in the steady state phase. We use this case study and the framework developed above to identify the potential ecological effects that might arise during the transitory and steady state phases. Our goal is to identify a range of effects that could potentially occur during the introduction of a GE insect. We derive these from the perspectives laid out in our framework described above. Although the epidemiology of malaria is relatively well understood, the ecological interactions of *An. gambiae* are poorly known. To use our framework to the fullest, we rely on information from other *Anopheles* species and other mosquito species to address these gaps in knowledge. Our effort below generates a comprehensive list of possible evolutionary and ecological effects of releasing GE *An. gambiae*, thus providing the foundation on which formal risk models, much broader in scope than currently used, can be developed to predict the likelihood and magnitude of potential ecological effects.

**Box 2. Using HEGs to suppress *An. gambiae***

A homing endonuclease gene, or HEG, is a type of selfish genetic element that exists naturally in fungi, bacteria, and plants, but has not been found in insects (Gould et al. 2006). A HEG is capable of inserting copies of itself onto homologous chromosomes that lack the genetic element. If such transmission occurs in the germline, the HEG can potentially propagate itself throughout an entire population (Gould et al. 2006). Theoretically, a HEG linked to a desired trait can spread rapidly through a population, even with fitness costs associated with the trait. Current research focuses on how HEGs may induce mortality when an individual is homozygous, thereby capitalizing on the HEG's ability to drive itself into a population while simultaneously suppressing the mosquito population (Deredec et al. 2011). Two ways HEGs are currently being researched to suppress mosquito populations are (1) by disrupting female fertility genes or, (2) by creating male-biased sex ratios. For the former, the HEG may insert itself into a vital fertility gene, thereby disrupting the gene and killing the developing offspring (Burt 2003). Homing may occur at a rate of 60% (Windbichler et al. 2011) and two to three HEGs are estimated to be needed for effective population suppression (Deredec et al. 2011). For a male-biased sex ratio, the HEG carried on the Y chromosome would cleave and disrupt the X chromosome (but not insert itself in this case), causing a greater number of males in the population (Windbichler et al. 2008). Implementation of either HEG method could suppress *An. gambiae* populations, ultimately reducing malaria transmission.

### Transitory phase

During the transitory phase, the *An. gambiae* population is expected to increase slightly because the release will supplement the existing population, and then decrease as the HEG spreads and suppresses the population. Deredec et al. (2011) showed theoretically that an introduction as small as 0.1% the population size can eliminate the *An. gambiae* population, which is likely within the range of typical population fluctuation given the high seasonal variation found in *Anopheles* species in other regions (Ndenga et al. 2006; Dantur-Juri et al. 2010). Therefore, we assume that the population increase will be negligible. However, we note that if this technology progresses to actual field trials, release rates and frequencies may be much higher than theoretically projected, and there could be nontrivial transitory population increases which we will consider at the end of this section.

#### Evolutionary effects

Gene flow from *An. gambiae* may affect other human disease-vectoring *Anopheles* species (Besansky et al. 1997). Matings between *An. gambiae* and both *An. arabiensis* and *An. merus* (all African endemics) may result in fertile offspring, and analysis of multilocus DNA polymorphisms suggests interspecific gene flow has occurred between wild populations of *An. gambiae* and *An. arabiensis* (Besansky et al. 2003). If interspecific transfer and introgression of the engineered gene(s) were to occur, even at low initial densities, this could suppress these other species, resulting in greater transient losses of acquired and herd immunity.

For HEG mosquitoes, *intra*specific gene flow is the means for successful vector suppression. Incomplete spread of the gene is particularly relevant to *An. gambiae* because it is a nominal species comprised of several different chromosomal forms (Nwakanma et al. 2013). If the HEG spreads to only some of the chromosomal forms, this may allow the others to increase in density, resulting in resistance to the HEG. The extent of reproductive isolation among forms is unclear; however, in at least some forms, frequency of hybridization among forms varies widely across different geographic regions (Nwakanma et al. 2013). Understanding the extent and distribution of hybridization and introgression among *An. gambiae* subpopulations is especially important for engineering the species.

Rapid evolution of resistance of *An. gambiae* to malaria management tactics (e.g., DDT (van den Berg 2009), pyrethroids (Ranson et al. 2011)) is an ongoing challenge (Webb 2011), and resistance to GE technologies could also occur. Resistance to a lethal HEG could evolve through increased prereproductive isolation between GE and non-GE mosquitoes. However, a recent single-generation study on GE-RIDL found no mating disadvantage for GE-RIDL *Ae. aegypti* males under semi-field conditions (Lee et al. 2013), which in turn suggests little selective advantage for prereproductive isolation. Resistance could also occur in *An. gambiae* if the HEG drive mechanism was self-limiting, creating alleles that cannot be converted to the lethal HEG. Alternatively, if the HEG driver disassociated from the linked lethal gene, the “unloaded” HEG might displace the linked lethal HEG (Gould et al. 2006).

#### Ecological interactions

Transitory changes in *An. gambiae* may be expected to affect interactions with other species. The transient effect of a rapid decline or extirpation of *An. gambiae* on other community members may not be different from the effects during the steady state phase, so these effects are detailed in the following section. However, if significant releases of HEG *An. gambiae* are required, despite theoretical predictions to the contrary (Deredec et al. 2011), this may result in other transient effects, such as a transient increase in prey for mosquito predators such as bats (Reiskind and Wund 2009), salamanders (Rubbo et al. 2011), fish, spiders, and various aquatic Coleoptera, Diptera, Hemiptera, and Odonata (Shaalan and Canyon 2009; and references therein). Furthermore, if the transitory increase is high, *An. gambiae* could increase competition with other mosquitoes. In an extreme case, competition for resources could lead to exclusion of other mosquito species (Carrieri et al. 2003). Though competition exclusion has yet to be documented in *Anopheles*, it has been documented in other mosquito genera (Juliano and Lounibos 2005). For instance, the invasive *Aedes albopictus* has reduced established *Ae. aegypti* population densities over the last two decades in North America (Juliano and Lounibos 2005).

The intended decline in the mosquito population resulting from any vector suppression tactic could result in a transient reduction in immunity in human populations. People living in areas with high malaria incidence acquire immunity after several bouts of malaria, which lessens the symptoms of the disease. These people remain infectious, and may lose their acquired immunity when they stop contracting malaria (Langhorne et al. 2008). When a malaria intervention reduces exposure to infective mosquitoes, prevalence of acquired immunity declines and human disease prevalence is initially predicted to decline, followed by a gradual long-term increase in disease. The long-term prevalence of disease may be higher or lower than preintervention levels (Ghani et al. 2009). In addition, acquired immunity can disrupt transmission (Bousema and Drakeley 2011), so loss of acquired immunity may increase transmission. Loss of acquired immunity has contributed to the failure of other malaria control tactics (Webb 2011), and could be a factor with GE mosquitoes, especially if vector suppression is only temporarily successful. For example, an insecticide-based malaria control campaign in Liberia resulted in a loss of acquired immunity in much of the target area, allowing a resurgence of malaria (Webb 2011).

### Steady state phase

Because the intended goal of releasing HEG *An*. *gambiae* is to suppress the vector population, a lowered population density in the steady state phase would occur if the technology is successful.

#### Evolutionary effects

The evolutionary effects from the transitory phase could persist into the steady state phase, and several new effects may occur as well. For example, the *Plasmodium* pathogen may evolve to resist the GE mosquitoes. Decline in the vector population might put added selection pressure on *Plasmodium* strains able to infect other mosquito species. Vector switching has been shown in other mosquito-vectored diseases: chikungunya virus has undergone adaptive mutations to switch to the vector *Aedes albopictus* from other *Aedes* species (Tsetsarkin and Weaver 2011). This may be particularly important if other potentially suitable mosquito vectors increase in number following *An. gambiae* decline, allowing for higher transmission rates and a resurgence of malaria.

Reduced *An. gambiae* populations could affect vector capacity of other mosquito species or of residual populations in the *An. gambiae* complex. Major factors influencing vector capacity in *Anopheles* are (1) the lifespan of the vector, (2) the amount of time needed for *Plasmodium* to develop from ingested gametocytes to transmissible sporozoites, (3) frequency of contact between mosquitoes and vertebrate hosts, and (4) the general susceptibility or resistance capability of the mosquito vector (Cohuet et al. 2010). Any of these factors could evolve in the other vectors; for instance, as *An. gambiae* decline in habitats surrounding human settlements, new species might colonize these spaces due to competitive release. Increased anthropophily is thought to be an important factor in the evolution of high vectorial capacity of *An. gambiae* (Cohuet et al. 2010). In fact, the malaria parasite is thought to have exerted selection on the behavior of mosquitoes to prefer human hosts with high parasite load (Lacroix et al. 2005).

#### Ecological interactions

The reduction of *An. gambiae* could have effects on ecological communities. The extirpation or decline of *An. gambiae* could allow for the dominance of *An. arabiensis*, a competitor whose range overlaps with *An. gambiae* and is also a vector for malaria (Kirby and Lindsay 2009). Dabire et al. (2012) showed *An. arabiensis* populations have increased over the previous 10 years as *An. gambiae* populations decreased, allowing *An. arabiensis* to thrive in urban areas once dominated by *An. gambiae* and achieve higher vector capacity.

The potential effects of *An. gambiae* removal on predators are not as clear. *An. gambiae* populations have been shown to be regulated in part by predation (Kweka et al. 2011), though the impact of adult *An. gambiae* removal on specialist predators is unknown. However, because mosquitoes often account for a significant portion of the diet of multiple generalist spiders, bats, and fish (USFWS, United States Fish and Wildlife Service 2005; Reiskind and Wund 2009; Manna et al. 2011), their reduction could subsequently reduce populations of these predators. This could cause cascading community effects, disruption of food webs, and the potential loss of diversity in the affected community.

The reduction of a mosquito species that performs a particular function in an ecosystem could result in a reduction of that function. While much work is still needed to fully describe their ecological roles, filtering of microorganisms and particulates by mosquito larvae can impact water chemistry (Power et al. 1996). Additionally, larvae can increase primary productivity through preferential grazing of phytoplankton and bacteria (Mokany 2007). Furthermore, specialist predators such as predaceous diving beetles feed on mosquito larvae and have evolved life cycles that closely mirror the life cycle of *Aedes* mosquitoes (Nilsson and Soderstrom 1988). Finally, some mosquitoes (e.g., *Toxorhynchites spp*. and *Psorophora spp*.) engage in intraguild predation and prey on the larvae of other mosquito species due to the ephemeral nature of their breeding grounds (USFWS, United States Fish and Wildlife Service 2005). While the effects of *An. gambiae* removal are unknown, it is plausible that removing these prey sources could have important consequences on the development and survival of specialist species.

Adult mosquitoes occupying terrestrial environments are hypothesized to be pollinators of angiosperms (Fang 2010). While many species of mosquitoes are attracted to flowers and feed on nectar (Müller et al. 2010), and a few species have been documented with pollen attached to their bodies (e.g., Gorham 1976), there is debate over whether mosquitoes provide pollination services or are simply nectar robbers (Fang 2010). We are unaware of research that has linked the absence of mosquito pollinators to an increase in plant pollen-limitation, but it is difficult to ascertain whether this absence of evidence is due to the absence of the functional role of mosquitoes or of a research bias. If mosquitoes do play a significant role in pollination, it is possible that their removal could cause pollen limitation and a decline in plant populations.

Finally, if the new steady state of a suppressed *An. gambiae* population was found to be undesired, could the system be reversed, or is it irreversible due to hysteresis? It is difficult to predict this from present ecological knowledge. If HEG mosquitoes reduce the prevalence of malaria as intended, this issue may become moot, but it should nonetheless be considered.

## Discussion

Researchers are rapidly developing many GE technologies. While GE technologies offer exciting possibilities for advancements in global pest and disease management, rigorous and concomitant assessments of potential associated risks are vital to ensure their full success. We have provided a framework to identify potential ecological effects of a GE insect release by examining the release as a two-phase process. Effects identified during the transitory phase dealt with gene flow within and between species, evolution of resistance, immunity, and transient changes in species interactions. Effects identified during the steady state phase dealt with evolution of interacting species and changes in ecological states. With these effect categories as a guide, we then applied this framework to identify potential ecological effects of GE mosquitoes to combat malaria.

A major advantage of our framework is its broad scope. Conceptualizing a release as an ecological perturbation of a population allows for utilization of a wide range of ecological studies to identify potential ecological effects of introduced GE insects. Moreover, separating the population change into transitory and steady state phases offers a more comprehensive method to identify potential ecological effects for a particular management strategy; it highlights how a *changing* versus a *changed* population can alter an ecological system differently. Effects in the transitory phase show how a perturbation may have immediate ecological effects, which is especially important if such effects do not persist into the final steady state. We illustrated this with our case study of HEG mosquitoes. For instance, if vector populations were suppressed, a reduction in acquired immunity could cause a transient increase in disease incidence (Bousema and Drakeley 2011), a phenomenon which is not necessarily unique to GE control strategies. Disease incidence may ultimately subside, but a transient increase could have significant implications for risk management and communication. Conversely, identifying effects occurring in the steady state phase highlighted effects that might result as the ecosystem adjusts to the changed population. For GE mosquitoes, this might include evolution of increased vector capacity (Cohuet et al. 2010), or knock-on effects through the ecosystem, which might harm valued ecological interactions and hinder efforts to suppress malaria.

Our framework also highlights important knowledge gaps. All ecological predictions are uncertain, but our approach partially compensates by pulling information from a wide variety of ecological sources. It also exposes areas that may be especially useful for directing future research. For instance, in evaluating GE mosquitoes, the knowledge gaps in mosquito ecology are striking, as noted by Godfray (2013), particularly with respect to mosquito effects on consumer and resource species. Data and theory on ecological hysteresis in insect communities are also lacking, which makes it difficult to assess whether any changes are irreversible. Furthermore, in our case study we found that all potential effects identified in the transitory phase were either transient effects with transient causes, or were persistently reinforced effects that continued into the steady state phase. Transient causes did not affect persistent steady state responses, but this could be a knowledge gap.

The use and release of GE insects is still in its infancy, and consequently, few precedents exist from which to draw examples of ecological effects. Past risk assessments concerning GE insect release (USDA, United States Department of Agriculture 2008; see Murphy et al. 2010 for assessment of *Wolbachia*-infected mosquito release) have identified ecological effects assuming successful steady state outcomes of the GE insect management tactic, while neglecting possible transient effects. We found that transitory phases may be nontrivial in duration and/or magnitude (e.g., Koyama et al. 2004). Analysis of the transitory phase allows for consideration of effects distinct from those in the steady state.

Finally, our framework provides a crucial step in the Problem Formulation phase of an ERA. Identifying the effects present in both the transitory and steady state phases will help risk assessors determine important ecological entities that require protection and establish causal pathways for effects on those entities (EPA, US Environmental Protection Agency 1998). Based on this, assessors and stakeholders can determine, using some socially acceptable process, whether the potential ecological effect is adverse and determine the scope of the risk assessment. Different societies may have different perspectives about which ecological effects may be adverse. Our systematic, synthetic framework for identifying potential ecological effects of GE releases allows for focused discussion on adversity and may help improve future risk assessments.
